#  Gastroschisis with Concomitant Jejuno-Ileal Atresia Complicated by Jejunal Perforation 

**Published:** 2016-04-10

**Authors:** Zlatan Zvizdic

**Affiliations:** Clinic of Pediatric Surgery, University Clinical Center Sarajevo, Sarajevo, Bosnia and Herzegovina

**Dear Sir**

Gastroschisis is an isolated structural anomaly rarely associated with other life-threatening anomalies. The most common associated gastrointestinal anomalies are intestinal atresia, Meckel's diverticulum and intestinal duplications. Intestinal atresia occurs in 10-15% of gastroschisis cases. [1] We herein report a case of gastroschisis with concomitant type III b jejuno-ileal atresia with perforation of the proximal dilated segment of jejunum with congenital short bowel successfully treated by primary anastomosis and primary abdominal closure.

A 7-hours-old male infant born to a healthy 20-year-old mother was referred to our institution for definitive treatment by the regional hospital. Baby was delivered by emergency Caesarean section due to fetal bradycardia at 36 weeks gestation. An Apgar score 1/5 was >7, whereas birth weight was 3200 grams. Family history was showed no case of gastroschisis. The clinical examination showed a gastroschisis with the entire stomach and the mid-gut herniating through the full thickness abdominal wall defect, just right of the umbilicus (Fig. 1). 

**Figure:**
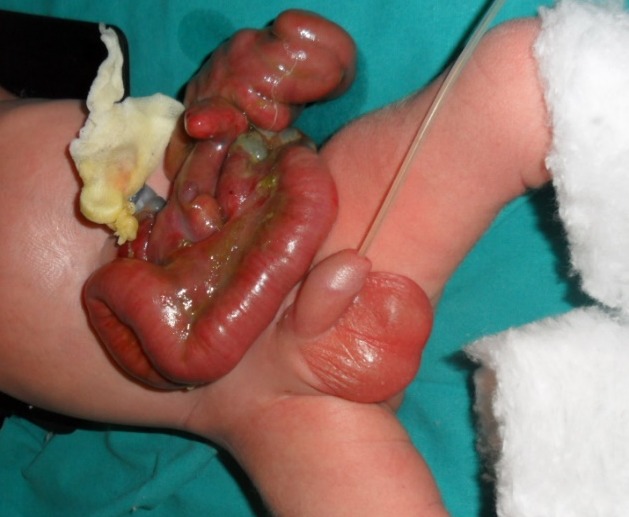
Figure 1: Gastroschis with concomitant jejuno-ileala tresia type III b.

No other severe congenital anomalies were detected. Initial resuscitation in the form of ongoing fluid correction and heat losses of exposed bowel and subsequent metabolic disturbance was conducted preoperatively. Perforation of the dilated loop of proximal jejunum which ended blindly was observed during the surgery (Fig. 2).

**Figure:**
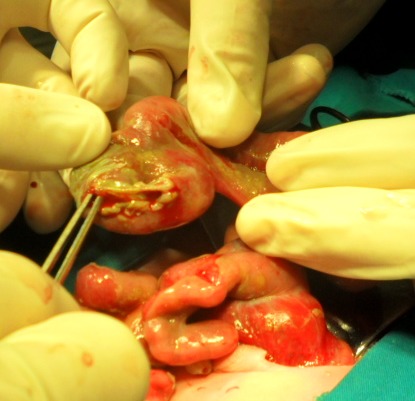
Figure 2: Intraoperative view of antenatally developed perforation of the dilated proximal jejunum.

Atresia of the jejunum was located 25 cm distal to duodeno-jejunal junction. Further exploration found ileal atresia 65 cm proximal to ileo-cecal valve. In our case of type III b jejuno-ileal or "apple peel" atresia, the total length of small intestine was 90 cm (25 cm distal to duodeno-jejunal junction and 65 cm proximal to ileo-cecal valve). Primary jejuno-ileal end to end anastomosis without liberal resection of the proximal bulbous jejunal segment and primary closure of the anterior abdominal wall with preservation of the umbilicus were performed. Postoperatively, patient was admitted in our NICU. The baby was treated with gastric decompression and total parenteral nutrition. After the restoration of enteral function, following the 13th postoperative day, low-volume enteral nasogastric tube feeding was initiated, with a gradual increase until full oral feeding was established in the twenty-sixth postoperative day. Precautionary, before each feeding gastric aspiration was performed. The baby was discharged on 31st day.

Treatment of patients with gastroschisis associated with intestinal atresia is a huge and serious challenge. The choice of adequate surgical treatment is controversial and largely depends on the assessment of reactive change of the intestine. Ideal treatment of such anomalies would consist of safe primary closure of the an-terior abdominal wall with the establishment of the intestinal continuity by primary anastomosis. Unfortunately, this scenario is rarely feasible. [2] Alternative treatment options may consist of primary, delayed primary, or staged silo closure of the anterior abdominal wall by leaving the atresia undisturbed or creating a stoma with the correction of intestinal atresia after few weeks. [3] Restoration of intestinal continuity by primary anastomosis with primary abdominal closure in our case was technically feasible and supported by parenteral nutrition in the postoperative course, all of which provided an excellent outcome. Even without the liberal resection of the proximal bulbous jejunal segment, in order to preserve the length of the proximal jejunum, intestinal peristalsis was established within time-frame that was not sig-nificantly longer than the establishment of peristalsis in patients with gastroschisis without intestinal atresia.

The management neonates with gastroschisis associated with intestinal atresia has improved in recent decades due to improvement of surgical technique, improved quality and organization of Neonatal Intensive-Care Unit (NICU), use of total parenteral nutrition (TPN) as well as the profiling of neonatal anesthesia as a separate sub-discipline. With the aforementioned appropriate treatment, the survival rate in infant with gastroschisis associated with intestinal atresia is higher than 90% but the average rate of morbidity in these patients is significantly higher.

## Footnotes

**Source of Support:** Nil

**Conflict of Interest:** None
